# Clinical and MRI outcome of cervical spine lesions in children with juvenile idiopathic arthritis treated with anti-TNFα drugs early in disease course

**DOI:** 10.1186/s12969-017-0173-1

**Published:** 2017-05-15

**Authors:** Damjana Ključevšek, Nina Emeršič, Nataša Toplak, Tadej Avčin

**Affiliations:** 1 Radiology Unit, University Children’s Hospital Ljubljana, Ljubljana, Slovenia; 2 Department of Allergology, Rheumatology and Clinical Immunology, University Children’s Hospital Ljubljana, Ljubljana, Slovenia; 3 Department of Pediatrics, Medical Faculty, University of Ljubljana, Ljubljana, Slovenia

**Keywords:** Juvenile idiopathic arthritis, Cervical spine arthritis, Magnetic resonance, Anti-TNFα, Outcome

## Abstract

**Backgrounds:**

The purpose of the study was to evaluate the clinical and magnetic resonance imaging (MRI) outcome of cervical spine arthritis in children with juvenile idiopathic arthritis (JIA), who received anti-TNFα early in the course of cervical spine arthritis.

**Methods:**

Medical charts and imaging of JIA patients with cervical spine involvement were reviewed in this retrospective study. Data, including age at disease onset, JIA type, disease activity, treatment and clinical outcome were collected. Initial and followup MRI examinations of cervical spine were performed according to the hospital protocol to evaluate the presence of inflammation and potential chronic/late changes.

**Results:**

Fifteen JIA patients with MRI proved cervical spine inflammation (11 girls, 4 boys, median age 6.3y) were included in the study: 9 had polyarthritis, 3 extended oligoarthritis, 2 persistent oligoarthritis and 1 juvenile psoriatic arthritis. All children were initially treated with high-dose steroids and methotrexate. In addition, 11 patients were treated with anti-TNFα drug within 3 months, and 3 patients within 7 months of cervical spine involvement confirmed by MRI. Mean observation time was 2.9y, mean duration of anti-TNFα treatment was 2.2y. Last MRI showed no active inflammation in 12/15 children, allowing to stop biological treatment in 3 patients, and in 3/15 significant reduction of inflammation. Mild chronic changes were found on MRI in 3 children.

**Conclusions:**

Early treatment with anti-TNFα drugs resulted in significantly reduced inflammation or complete remission of cervical spine arthritis proved by MRI, and prevented the development of serious chronic/late changes. Repeated MRI examinations are suggested in the follow-up of JIA patients with cervical spine arthritis.

## Background

Cervical spine arthritis is a well-recognized complication of juvenile idiopathic arthritis (JIA), usually presenting in patients with systemic or polyarticular forms of the disease [1]. More commonly, the upper cervical spine is affected because the atlanto-occipital and the atlanto-axial joints are synovial and are thus primary targets for rheumatic inflammation [2]. Early detection of cervical involvement is essential for identification of children that require more aggressive treatment including early introduction of biologic therapy. Untreated chronic inflammation of the cervical joints can lead to morphological changes and functional impairment, which can be even life-threatening due to instability and potential risk of spinal cord injury.

Clinical signs and symptoms of cervical spine arthritis may include neck pain, stiffness, torticollis, limited range of motion (LROM), headache, facial and ear pain, and signs of cervical myelopathy. Absence of clinical symptoms does not exclude subclinical inflammation and imaging abnormalities of cervical spine [3].

Imaging modalities used in assessment of cervical spine arthritis include radiography, computer tomography and magnetic resonance imaging (MRI) [4–6]. Radiography has been most evaluated imaging modality for assessment of cervical spine lesions and is particularly useful for evaluation of malalignment, functional impairment, morphological changes of bones, and vertebral body growth disturbances [3, 7–9]. In general, MRI is the method of choice in evaluation of cervical spine arthritis and is most useful in detecting synovitis with hypertrophic, enhanced synovia and pannus formation, joint effusion, erosions, bone marrow edema and possible neural structure compressions [10, 11]. The diagnostic accuracy and predictive value of conventional MRI in JIA patients with axial joint involvement have been poorly investigated [12]. The role of MRI in detecting early changes in cervical spine in JIA was first evaluated in a Turkish study showing higher sensitivity of MRI compared to radiography for detection of erosions in children [13]. More recently, a German study evaluating MR changes of cervical spine arthritis at diagnosis and response to treatment in 13 patients with JIA was published [14]. It was concluded that close MRI monitoring of these patients appears to be sensitive tool for early diagnosis and may help to detect further disease progression and complications.

In the last two decades, highly effective biologic therapies became available. The efficacy of treatment with anti-TNFα should be assessed and validated by standardized imaging protocols for data acquisition and interpretation. MRI scoring for patients with rheumatoid arthritis is a good alternative, but it was not adapted and validated for joint involvement in children with JIA [15]. The main problem in assessment of arthritis in children and adolescents are difficulties to differentiate between physiologic and pathologic joint findings [16–18]. Therefore, there is a clear need for development of tools for more accurate monitoring of therapeutic responses for both disease activity and structural damage to the joint [15].

According to ACR 2011 Recommendations, cervical spine arthritis was considered a poor prognostic factor that may require escalation of therapy [19]. We decided to introduce anti-TNFα treatment in all children with proven cervical spine arthritis on MRI [20]. Therefore, the objective of our study was to evaluate the clinical and MRI outcome of cervical spine arthritis in a group of consecutive patients with JIA who received anti-TNFα treatment early in disease course. Our preposition was that treatment with anti-TNFα early in the course of cervical spine arthritis could provide a good response to active inflammation with potential less chronic changes.

## Methods

The study was designed as a retrospective cohort study with longitudinal follow-up of clinical and MRI data in children with JIA. The charts of 407 children with JIA treated at the University Children’s Hospital between September 2010 and July 2016 were reviewed and all children with cervical spine involvement confirmed by MRI examination were included in the study.

In all children clinical signs of neck involvement (LROM, neck pain, stiffness and torticollis) and involvement of other axial and peripheral joints were assessed at the time of initial presentation of cervical arthritis and at their last follow-up visit. MRI of cervical spine was performed in all children within 3 weeks of clinical notion of cervical spine involvement and evaluated according to the hospital protocol. All MRI examinations of cervical spine included at least one short Tau inversion recovery (STIR) (usually in coronal or sagittal plane), T2 TSE and T1 TSE in sagittal and axial plane, T1 TSE FS axial, and postcontrast sequences T1 TSE FS in 2 or 3 planes (Magnetom Aera or Avanto 1.5 T MR Siemens Healthcare). MRI evaluation of cervical spine consisted on determination of synovitis, bone changes and the presence of malalignment. Synovitis was evaluated as presence of synovial thickening in cervical spine (predental and paradental space of atlanto-axial joint, atlanto-occipital joints and facet joints), presence of fluid in these joints, and evaluation of ligaments in this region (transversus, alar and apical ligaments). Enhancement of synovia and joints after i.v. administration of paramagnetic contrast medium was determined. Bone evaluation consisted of presence or absence of bone edema and morphologic changes of bone (dens deformation, thinned corticalis of bone in involved region, erosions, ankyloses). Malalignment was evaluated as abnormal position of articular surfaces of atlanto-occipital (basilar invagination), atlanto-axial (anterior atlanto-axial subluxation - aAAS), facet joints (subaxial subluxation), and narrowing of craniocervical spinal canal. In children with suspected malalignment radiography of cervical spine in antero-posterior and lateral position was performed.

Data about therapy including the type of medication and time from disease onset and treatment introduction was collected for all patients.

Follow-up examinations included clinical evaluation and serial follow-up MRI examinations of cervical spine. The timeframe of follow-up MRI examinations depended on the severity of cervical spine inflammation found on the initial MRI and treatment efficacy. Persistent clinical signs (more than 3 months) were indication for earlier MRI follow-up. If at least three compartments of AA and AO joints were involved, or additional facet joints inflammation, in case of abounded pannus resulting in malalignement, and presence of bone edema even in the case of good clinical response, follow-up MRI was performed 5 to 6 months after the initial one to make an objective evaluation of treatment response. In other children the first follow-up MRI was performed 9 to 12 months later. On average, patients had 2.4 (range 1–8) follow-up MRI examinations with mean time interval between the initial and the first follow-up examination 6.5 months (range 2 to 14 months) and between initial and the last MRI examination 27.9 months (range 6 to 71 months). Initial and follow-up MRI findings were compared and the response to therapy was determined as no response (same), partial improvement (reduced inflammation compared to previous MRI), and no inflammation (no MRI signs of active inflammation or minimal residual patchy enhancement). Serial MRI examinations enabled not only evaluation of treatment response, but also evaluation of the development of chronic bone changes during the observation period.

In addition, suspected malalignment was assessed on cervical radiography in antero-posterior and lateral projections in 3 children. Cervical radiography in antero and retroflexion positions was performed at the time when the patient had no signs of active inflammation.

Descriptive statistics was used to summarize the study.

## Results

### Initial presentation of cervical spine arthritis

Cervical spine involvement was identified in fifteen out of 407 (3.7%) children with JIA (4 boys and 11 girls) ranging in age from 2.5 to 15.1 years (mean 6.3 years). Eight children were diagnosed with rheumatoid factor (RF) negative polyarthritis (pJIA), 1 with RF positive polyarthritis, 3 with extended oligoarthritis (oJIA), 2 with persistent oJIA, and 1 with juvenile psoriatic arthritis (jPsA).

Cervical spine involvement occurred in 9/15 (60%) children within the first 6 months from the disease onset including 3 patients with cervical arthritis as the initial manifestation of JIA. Six out of 15 patients (40%) developed cervical spine arthritis more than 1 year after the disease onset. Mean disease duration at the time of diagnosis of cervical spine arthritis including 14 patients in our cohort was 5.2 months (range 1 month to 13 months), excluding the patient #11, who presented with cervical arthritis 13.5 years after the disease onset. All children clinically presented with LROM, two of them had stiff neck and one torticollis. Twelve children complained about the pain in the neck. None of our patients developed cervical spine involvement as the isolated manifestation of JIA. Mean number of active peripheral joints at the time of diagnosis of cervical spine arthritis was 18.1 (range 2 to 40 active peripheral joints). Three out of 15 patients (20%) had signs of temporomandibular joint (TMJ) arthritis together with cervical spine arthritis. One patient had associated uveitis.

Laboratory examinations at the time of presentation revealed normal erythrocyte sedimentation rate (ESR) in 2 and elevated ESR in 13 patients with the mean value of 36 mm/h (range 9 to 77), mean white blood cell (WBC) count 8.9 × 10^9^/L (range 5.7–19.3), mean hemoglobin109 g/L (range 92–133) and mean platelet count 457 × 10^9^/L (range 109–782). Ten out of fifteen (67%) patients had positive antinuclear antibody (ANA) titres. Only one out of 11 patients had positive human leukocyte antigen (HLA) B27. Fourteen patients had negative RF and one patient (#12) was RF positive.

Initial MRI examination demonstrated in all children atlanto-axial synovitis with enhanced synovia during i.v. application of paramagnetic contrast medium, 9 had also fluid in predental space. Co-existed atlanto-occipital synovitis was found in 6 children, and additional fluid in joint space was found in 3. Involvement of facet joints was seen in 8 children including one child with 6 affected facet joints. Bone edema of masse lateralis and thickened ligaments were found in 2 children. Anterior atlanto-axial subluxation at presentation was seen in one girl (patient # 8). No morphological changes of bone (cortical thinning or dens deformation) or erosions were seen in the initial MRI examinations.

All children were initially treated with high-dose intravenous pulse methylprednisolone therapy followed by oral corticosteroid and methotrexate (MTX, 10-15 mg/m2/once per week per os). Eleven out of 15 (73.3%) patients were additionally treated with anti-TNFα drug within 3 months of clinical signs of cervical spine involvement confirmed by MRI (6 with infliximab, 3 with etanercept, and 2 with adalimumab). There was a delay in introduction of biological treatment in 3/15 children. The delay of 4 months between the initial sign of cervical spine involvement and treatment with biologics in one patient (#14) was due to parents concerns regarding the safety of biological treatment. Two patients in our cohort (#12 and # 13) were initially treated only with corticosteroids and MTX according to the decision of the treating physician and received treatment with infliximab 6 months after the beginning of cervical spine involvement due to unsatisfactory results with initial treatment seen in the follow-up MRI. Both patients were later treated with infliximab and achieved a sustained remission. One patient (#15) from our cohort was treated only with MTX and pulse methylprednisolone therapy and did not receive biologics due to parent refusal. This patient also achieved inactive disease state.

### Follow-up

The mean observation time from diagnosis of cervical spine arthritis to July 2016 was 2.9 year (from 0.5 to 6.8 years).

At the last follow-up examination 4 children had still some cervical LROM, but all children were without pain, torticollis or neck stiffness. Peripheral joint arthritis persisted only in 4 patients with 1 to 5 active peripheral joints. ESR significantly decreased to mean value of 11 mm/h (range 4–25 mm/h). Decreases were also noted in the mean value of WBC (7.3 × 10^9^/L (range 2.9–11.7)) and mean platelet count (308 × 10^9^/L (range 128–411)), while hemoglobin level increased to the mean value of 123 g/L (range 107–141)).

Mean duration of treatment with biologics in our cohort was 2.2 years (range 0.5 to 4.5 years). In three patients treatment with biologics was stopped after a mean period of remission on treatment for 3.4 years (range 2.1 to 4.5 years). After stopping treatment with anti-TNFα two patients remained in sustained remission without any treatment for more than 2 years, and one patient continued treatment with MTX. A girl with jPsA (patient # 7) who stopped treatment with biologics after 2.1 years remained in remission without treatment for 2.3 years, and then presented with reactivation of cervical spine arthritis with MRI signs of synovitis of atlanto-axial, atlanto-occipital and right C2-C3 facet joints. She was retreated with infliximab and again achieved a stable remission for 1.5 years.

**Fig. 1:**
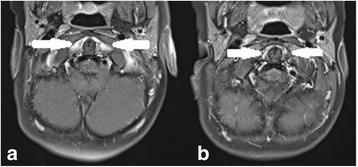
MRI of cervical spine (patient #5) (T1 TSE FS axial with a contrast medium): **a** inital MRI showed an intense contrast enhancement of the thickened synovia in pre- and paradental space (white arrows), **b** follow-up MRI 6 months later showed a reduction of synovial thickening and a significantly less intense enhancement (white arrows)

**Fig. 2:**
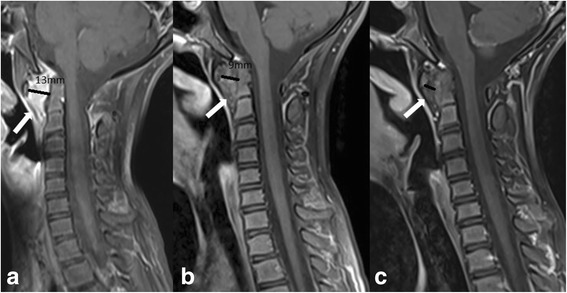
MRI of cervical spine (patient #8)(T1 TSE FS sagittal with a contrast medium): **a** initial MRI (January, 2013) showed an intense contrast enhancement of a huge synovial thickening (white arrow) in predental space with a huge anterior atlanto-axial subluxation (aAAS)(black line, 13 mm) and a compression of the anterior liquor space, normal shape of the dens, **b** follow-up MR (January, 2014): the evaluation of the treatment with anti-TNFα showed a reduction of synovial thickening and a less intense contrast-enhancement and less significant aAAS, normal width of the anterior liquor space, but with initial morphological changes of the dens, **c** follow-up MRI (March, 2016): the evaluation of the treatment with anti-TNFα showed a significant reduction of synovial thickening in the predental space and aAAS, only patchy contrast enhancement, but with morphological changes of the dens and a thinned corticalis

The last follow-up MRI revealed no signs of inflammation in 12 patients (6 with no signs of arthritis and 6 with minimal residuals in a form of minor patchy enhancement of cervical synovia, which could be considered as normal), in 2 patients improvement of arthritis, but with MRI signs of persistent active inflammation (remnants of synovial thickening with moderate contrast enhancement in patients # 5 and # 6, both received anti-TNFα for 6 months)(Fig. 1), and in one significant improvement of arthritis, but with mild to moderate MRI signs of active inflammation (patient#8) (Fig. 2).

Chronic changes (late sequels) in cervical spine due to inflammation were seen in 3 patients at the last follow-up MRI examination. Two of them (patients #12 and #15) had minor chronic changes in a form of irregular bone contour of dens and thinned corticalis. In addition, the girl with RF positive polyarthritis (patient #12) had thickened transverse ligament not compromising the spinal canal, and the boy (patient #15) had borderline aAAS (5 mm) without additional increase of the distance during functional radiography of cervical spine. More severe chronic changes were found in a girl (patient #8) who initially presented with severe inflammation in upper cervical spine and significant a AAS (13 mm), narrowing of spinal canal at this level with compression of the spinal cord at the initial MRI (Fig. 2a). At the last follow-up MRI the aAAS reduced to 6 mm with remnants of thickened ligament in anterior atlanto-axial space and morphologically deformed enlarged dens with bulging dorsal contour (Fig. 2b). There were no signs of chronic changes in other children.

## Discussion

Cervical spine involvement in children with JIA is considered a poor prognostic factor and could be life threatening [19]. Therefore, obtaining a good insight into its frequency, clinical and imaging presentation is of vital importance.

According to published data, symptoms and signs correlate poorly with the degree of cervical spine inflammation and possible subluxations. In adult population, cervical spine involvement is seen in 35–65% of patients with JIA [3]. This wide range of reported cervical spine involvement in adult patients with juvenile-onset idiopathic arthritis could, to some extent, reflects recruitment bias since some studies were focused on clinical manifestations and others on radiographs or MRI changes [3, 9, 21, 22]. In our study, all children had LROM, 80% had neck pain, two neck stiffness and only one torticollis. These findings are in line with the German study suggesting that the LROM in cervical spine and not pain is a leading clinical sign of cervical spine arthritis [14]. The frequency of cervical spine involvement in our cohort of patients with JIA is estimated to be 4%. It is difficult to compare epidemiological data with other prevalence studies, because the duration of observation of patients in different studies varied or the studies were conducted only in selected JIA subtypes such as polyarticular and systemic forms of the disease. It is also important to note that different treatment modalities and protocols were used in the era before biologics, and more aggressive treatment, which is being used recently, may prevent some symptoms to develop. In addition, our study was conducted only in patients with cervical spine symptoms and some asymptomatic patients may have been missed.

A significant clinical observation in our study was that all children had peripheral joints involvement and in addition, 20% of them had co-existing unilateral or bilateral TMJ arthritis. The TMJ MRI examinations from our patients with TMJ involvement showed severe TMJ inflammation in all cases with intensive synovitis, joint fluid and significant morphological changes of mandibular condyles. In only few TMJ MRI examinations atlanto-axial joint was seen on T2 coronal sequence and we found in one patient signs of inflammation in atlanto-axial joint without clinical complains of pain and LROM. To identify patients with TMJ arthritis and possible clinically covert cervical spine involvement we suggest to include the atlanto-axial joints in coronal plane also on standard TMJ MRI examinations.

Imaging in patients with JIA is particularly important for joints that are difficult to assess clinically such as hip, sacroiliac joint and spine. In adults radiography of the cervical spine is accepted as mandatory in rheumatoid arthritis patients with neck pain and it is well standardized. On the other hand, studies reported low sensitivity of radiography in children because it provides morphological and structural information more typical for late phase of inflammation (dens deformation, erosive changes) and various forms of malalignment and ankylosis, which are rare in children as a first manifestation of cervical spine arthritis [13, 23]. At least in the initial stage cervical spine radiography is not a routine imaging in children, but it can be useful in children with malalignment and persistent changes seen on MR to obtain functional information of cervical spine. The most frequently noted abnormalities seen on cervical radiography are apophyseal joint ankyloses or fusion at C2-C3, followed by aAAS, subaxial subluxation at levels between second and seventh cervical vertebrae, and erosions of the dens resulting in an “apple core” deformity [9]. Atlantoaxial impaction (AAI) is another serious complication of longstanding rheumatoid arthritis, rarely seen in childhood. In our cohort radiography of cervical spine was performed in 20% of children to evaluate the malalignment and morphological changes of bones due to persistent LROM or signs of aAAS presented on MRI. Contrast-enhanced MRI of cervical spine has become the method of choice in evaluation of inflammation in this region [11, 14]. Standardization of MRI protocol enables more reliable follow-up examinations and comparison of initial MRI with controls [15, 24]. The main advantage of MRI is its ability to detect early, often subclinical disease by directly visualizing not only soft tissues changes in a form of synovial thickening/enhancement, joint effusion, facet joints inflammation, and ligament evaluation, but also bone changes in a form of edema, cortical thinning, morphological changes (especially dens deformation, evaluation of bone erosions), and malalignment. The presence of bone marrow edema was recognized as a strong predictor of future erosions and it is considered as pre-erosive abnormality in adults and indication for treatment initiation to prevent permanent joint damage [25]. Early detection of joint pathology is of outmost importance with regards to the prompt therapeutic intervention. It is important to note that normal synovia in childhood can provide some degree of enhancement, and that there are no imaging criteria to clearly distinguish normal findings from early signs of arthritis. Moreover, postcontrast MRI should be performed during first 5–10 min of injection, because beyond this time diffusion of contrast material into the joints limits differentiation between enhancing synovia and adjunct joint fluid [26]. Rheumatoid arthritis MR imaging score (RAMRIS) has been shown to have acceptable applicability to children in evaluation of the inflammation and response to treatment despite differences in the pediatric and adult skeletons, but was primarily designed for peripheral joint and not for assessment of cervical spine [27, 28]. Synovitis of atlanto-axial joints was present on MRI in all our patients, and more than half of them had synovitis in other cervical joints compartments and facet joints. The degree of inflammation presented on MRI varied from mild (unilateral atlanto-axial inflammation) to moderate (bilateral inflammation) (Fig. 1) and severe (extensive pannus in atlanto-axial region)(Fig. 2). The early introduction of anti-TNFα in treatment of JIA patients with severe form of the disease provides an opportunity to delay or even prevent severe joint inflammation and subsequent destruction [20, 29]. MRI as objective imaging method has important role in evaluation of treatment response. The evaluation of early treatment with biologics is usually done by comparing initial and follow-up MRI examinations in each child. No official scoring system for MRI evaluation of cervical spine arthritis in children is available [15]. The only MRI based follow up study of cervical spine arthritis in children treated with anti-TNFα was done by Hospach et al. [14]. The cohort of patients in this study was comparable to our patients and included 13 children, 12 of whom were treated with biologics, but with longer median disease duration of 1.7 years after the diagnosis of JIA (in our cohort 5.2 months) and shorter observation time. A variable duration of treatment with MTX and biologics has not been specified. Our study was in concordance with the abovementioned study in evaluation of inflammation, and showing a significant reduction of MRI signs of inflammation in cervical spine. The most significant difference between the study of Hospach et al. and our study was the evaluation of “chronic/late” changes. The follow-up MRI examinations in the study of Hospach et al. described more chronic changes in a form of malalignment in 3 patients after treatment, ankyloses in 3, erosion in 2, and narrowing of spinal canal in 3. In our study, half of the children with delayed biologic treatment had minor chronic changes compared to children with early introduction of biologics, where only one girl (#8) with severe and persistent inflammation developed more severe chronic changes, including dens deformation with bulging into spinal canal, thickened transverse ligament and persistent aAAS (Fig. 2). The low rate of long term consequences in our study could be due to earlier recognition with shorter disease duration and early aggressive treatment with biologics.

In 3 children we were able to withdraw the anti-TNFα after 2.1, 3.6 and 4.5 years, respectively. The duration of treatment depended on clinical signs of arthritis not only in cervical spine, but also in peripheral joints. In one patient (#7) disease relapse occurred after 2.3 years of inactive disease with a good response to reintroduction of anti-TNFα treatment. In 4 children the anti-TNFα was changed due to disease relapse in peripheral joints or unsatisfactory treatment result of cervical inflammation; two of our refractory JIA patients received also anti-IL-6 therapy as specified in the Table 1.

**Table 1:** Clinical and MRI examination data in patients with cervical spine involvement

	G	JIA	iMRI age (y)	Biologics	Treatment duration with biologics(y)	last MRI results		Clinical outcome
						Inflam	Chronic changes	
1	M	pJIA ANA+	4.1	ETA ADA	0.7 1.3	no	no	no inflam.
2	F	pJIA	7.6	IFX	1.8	no	no	no inflam.
3	F	pJIA ANA+	6.9	ETA	1	no	no	no inflam.
4	F	oJIA	3.2	ETA	2.8	no	no	no inflam.
5	M	pJIA ANA+ HLAB27+	2.4	IFX	0.5	peristent	no	Inflam. in 2 perif.joints
6	M	pJIA	14.8	IFX	0.5	persistent	no	LROM Inflam. in 5 perifer.joints
7	F	jPsA	3.6	IFX	2.1	no	no	no treatment for 2.3y
			8.2	IFX	1.5	no	no	LROM no inflam.
8	F	pJIA ANA+	8.7	IFX	3.5	peristent	yes	LROM Inflam. in 2 perif.joint
9	F	oJIA ANA+	15.1	IFX	1.2	no	no	no inflam.
10	F	pJIA	2.7	ADA TOC ETA	0.3 1.3 1.7	no	no	LROM no inflam.
11	F	oJIA	14.6	ADATOC	0.4 0.8	no	no	Inflam. in 1perif.joint
12	F	poJIA ANA+ RF+	3.1	IT IFX	0.5 4.5	no	yes	no inflam. uveitis noanti-TNFα for 2y MTX
13	F	poJIA ANA+	4.8	IT IFX	0.6 3.6	no	no	no inflam. no treatment for 2.2y
14	F	pJIA	2.5	IT ETA ADA	0.3 0.5 0.4 2.4	no	no	no inflam.
15	M	pJIA ANA+	4.2	IT MTX only	1.5 2.5	no	yes	no treatment for 1.4y

*G* gender, *F* female, *M* male, *pJIA* polyarticular juvenile idiopatic arthritis, *oJIA* extended oligoarthritis JIA, *poJIA* persistent oligoarthritis JIA, *jPsA* juvenile psoriatic arthritis, *iMRI* age of child when initial magnetic resonance imaging of cervical spine confirmed cervical spine arthritisBiologics, *IFX* infliximab, *ETA* etanercept, *ADA* adalimumab, and Anti IL-6, *TOC* tocilizumab, *IT* initial treatment with corticosteroids and methotrexate (MTX) received all children, duration of IT was written only in children with delay introduction of biologics Last MR: Inflam – no inflammation, persistent inflammation of cervical spine seen on last MRI, chronic changes - no chronic changes, yes- chronic changes seen on last MRI Clinical outcome: no inflammation – no inflammation in other peripheral joints, X periph.joints- number of peripheral joints presented with inflammation

Limitations of our study include the retrospective design and small cohort of patients. Additional limitation is also the lack of a real gold standard in determination of the severity of neck arthritis. In spite of the limited number of patients, our results demonstrated that early and aggressive treatment of cervical spine arthritis with anti-TNFα could provide good results with elimination of inflammation (early response) and has a potential to reduce/deminish the chronic changes. According to our experience, in all children with persistent LROM MRI should be performed and if inflammation is confirmed, anti-TNFα treatment should be considered [20]. The follow-up MRI examinations are individually addressed. However, according to our and others experiences [14], we suggest the first follow-up MRI after 9 months, and if the clinical and laboratory responses are satisfactory, the second follow-up MRI should be performed 1 year later to evaluate possible development of chronic/late sequels. If the clinical signs of cervical arthritis persist, MRI should be performed earlier (after 6 months). Due to high incidence of co-existing TMJ inflammation (20% in our cohort), we suggest that the transversal plane during MR examination of cervical spine include TMJ (at least one of the fluid sensitive sequences). On the contrary, in children with MRI examination of TMJ atlanto-axial joint should be included in one of the fluid sensitive coronal sequences.

## Conclusions

In conclusion, in children with JIA and cervical spine involvement the early treatment with anti-TNFα drugs showed good results evaluated with follow-up MR examinations. Prompt treatment led to significantly reduced inflammation or complete remission, and prevented the development of chronic/late changes. To determine the prevalence and severity of chronic changes in patients with early cervical arthritis treated with “biologics” further long term studies should be performed in larger population of JIA patients.

## Abbreviations

aAAS: Anterior atlanto-axial subluxation
ANA: Antinuclear antibody
ESR: Erythrocyte sedimentation rate
HLA: Human leukocyte antigen
JIA: Juvenile idiopathic arthritis
jPsA: Juvenile psoriatic arthritis
LROM: Limited range of motion
MRI: Magnetic resonance imaging
MTX: Methotrexate
oJIA: Juvenile oligoarthritis
pJIA: Juvenile polyarthritis
RAMRIS: Rheumatoid arthritis MR imaging score
RF: Rheumatoid factor
STIR: Short Tau inversion recovery
TMJ: Temporomandibular joint
WBC: White blood cell
